# Reactance, Decisional Procrastination, and Hesitation: A Latent Class Analysis of Clutter Behavior

**DOI:** 10.3390/ijerph20032061

**Published:** 2023-01-23

**Authors:** Devki A. Patel, Verena Graupmann, Joseph R. Ferrari

**Affiliations:** Department of Psychology, College of Science and Health, DePaul University, Chicago, IL 60614, USA

**Keywords:** procrastination, psychological reactance, clutter

## Abstract

During the 2019–2020 global pandemic, mandated time at home suggested engagement in personal projects, such as home decluttering. Clutter (an overabundance of possessions) may impede one’s quality of life by interfering with space livability and social connections and prompting negative affect and financial problems. The present study explored action–state orientation, psychological reactance, and decisional procrastination with 227 American adults (*M* age = 49.9 years old). A latent class analysis tested for differences in cognition across groups. Results supported that persons who struggle with clutter reflect clusters or “classes” given their self-reported cognitive processes, with hesitant and indecisive participants experiencing greater negative impacts of clutter than action-oriented and decisive participants. Our findings suggested improving the decision-making and goal-directed capacities of those struggling with clutter may be effective as preventive and/or interventive strategies. Future research might consider when hesitation to initiate challenging tasks and indecision emerge in the decluttering timeline and how those who are prone to these cognitions may be supported in managing their personal possessions.

## 1. Introduction

The overaccumulation of physical, personal possessions, usually termed clutter, often becomes disorganized [1]. Clutter impacts the utility and livability of one’s home, and one’s psychological well-being [2]). Past research examined clutter’s effects on decision-making, life satisfaction, and well-being, uncovering more indecision, reduced life satisfaction, and worse overall well-being because of clutter [2,3,4,5]. Several studies discussed the impacts of clutter on specific measurements of well-being, showing positive relationships between clutter and lower quality of life, stress, binge eating, and increased work-related tension [6,7,8,9]). Negative impacts of clutter appear to affect people across the lifespan, reducing subjective well-being in university students, adults, and older adults [5,10,11]. Together, these studies suggest the greater the amount of home (or, even office) clutter, the lower one’s reported life satisfaction, productivity, and quality of life.

The process of over-accumulating items varies greatly because of individual personality differences, situational factors, and past disposition and disposal behavior [9,12]. Examples of individual-level factors which may predict the impact of clutter on our lives include the acceptance or rejection of social norms around clutter and one’s procrastination tendencies. Much of the existing research on clutter focuses on clinical samples exhibiting symptoms of hoarding [13,14].

Hoarding disorder is distinguished from cluttering behavior in several ways. Normative collecting or cluttering is structured, deliberate, and pleasurable, while hoarding is unstructured, unplanned, and distressing [15]. Certain groups may be more likely to experience difficulties decluttering to manage their personal possessions. While hoarding is distinct from cluttering behavior, persistent failure to declutter is considered hoarding behavior and is an antecedent of hoarding disorder [16]. In one study, higher rates of hoarding behavior were observed amongst participants who were women, unmarried, and living alone [17]. Amongst people diagnosed with hoarding disorder, physical clutter was associated with impaired livability, interference with hygiene, and according to the authors, posed a very serious threat to older populations who experience increased health-related issues. The causes of hoarding disorder are unclear; however, deficits in social and occupational functioning are known to contribute to hoarding disorder [18]. The diagnosis of hoarding disorder includes a specification for excessive acquisition, which is defined as the accumulation of items that are not needed for which there is no available space [6]. More than eighty percent of individuals with hoarding disorder display excessive acquisition [14].

Surveying of consumption patterns in the general population often occurs through market research, which is usually not publicly available [19]. Market research tends to focus on decision-making at the acquisition stage and rarely considers post-consumption behavior such as disposal. Thus, our collective understanding of how overconsumption and overaccumulation interact with cognitive processes is still limited. A survey conducted by the buying and selling app Mercari reported an average of 42 unused items amounting to USD 723 in the average American household, with more than 50% of participants feeling overwhelmed by the amount of “stuff” in their home [20]. Although most Americans admit to having issues with the unused items in their homes, research in the area has focused primarily on the initial consumer behavior, the acquisition of items, rather than the cognitive processes behind disposal behavior, or decluttering.

The proposed research will study decluttering behavior in a community-based sample, yielding insights on how and why decluttering outcomes vary in people who know they are struggling with managing their personal possessions but are not diagnosed with hoarding disorder. Research on clutter in non-clinical samples is sparse; however, there is a consensus that decluttering is a skill and can be learned [6]. Exploratory analyses, though limited in their generalizability, open avenues of future experimental research. The roles of psychological reactance, indecision, and action–state orientation are considered as they related to decluttering outcomes.

Psychological reactance may explain why external pressures to declutter lead to undesirable outcomes. The theory of psychological reactance suggests our motivation changes when the freedom to act freely is threatened or lost to resist against the loss of freedom [21]. This includes resistance against social influence or infringement of personal space or privacy. Cross-cultural research on psychological reactance in response to threats to freedom cross-culturally showed the specific role of self-construal in how a threat to freedom is linked to reactance as well as on the relevance of taking into account psychological reactance in persuasive communication [22,23,24].

Reactance is observed in a wide range of contexts, such as underage drinking and risky behavior, health education, advertising, and littering [25,26,27,28]. More recently, reactance was shown to be elicited by controlling parenting behavior and predicted internalizing and externalizing problems in adolescents [29]. In a study of smart home energy management systems, researchers examined how the type of language used and justification given by the smart thermostat in a phone notification affected reactance [30]. Authoritative language and the lack of justification for temperature change was significantly more likely to induce reactance, confirming the crucial role of communication in the experience of reactance. 

Reactance theory helped to explain why efforts to prevent the spread of COVID-19 vary from individual to individual. A study of restaurant customers and their reactions to COVID-19 prevention measures found that individuals perceived normative appeals of COVID-19 prevention differently and that reactance played a role in how these prevention efforts were accepted or rejected. Consumers in this study were more concerned with the content of COVID-19 prevention messages than they were with the tone of the message itself; they were more likely to experience reactance to injunctive normative appeals over descriptive normative appeals [31]. Another study found greater perceived freedom threat was associated with greater reactance, which predicted lower intent to comply with COVID-19 precautions [32].

At the time of data collection for the present study, consumers were experiencing both resource scarcity and intense government mandating, suggesting a public experience of reactance [33]. An advertising study explored forced viewing of “pop-up ads” and intrusiveness during task completion, finding that the intensity of cognition at the time of forced viewing predicted feelings of intrusiveness [27]. The experience of reactance requires the perception of threat and is accompanied by emotions such as discomfort, hostility, aggression, and anger [34]. Despite having good intentions, family members who pressure partners or their children to declutter may be eliciting reactance as opposed to motivating decluttering behavior.

The spaces we call “ours”, such as bedrooms, are often seen as extensions of one’s self-identity [35]. Imposing decluttering or disposal of personal possessions may feel threatening, especially if the individual has strong attachments to their items [5]. The experience of reactance in decluttering projects may go beyond causing negative emotions, as forceful or persistent messaging may complicate decision-making processes and hinder decluttering altogether.

Procrastination is a decision-making strategy employed to maximize short-term benefits at the expense of a later commitment or requirement [36]. All forms of procrastination are maladaptive, with negative impacts on the quality of life for residents of both individualized and industrialized countries [37,38]. Decisional procrastination, also called indecision, may become habitual and lead to passive procrastination, a persistent tendency to delay decisions and important work [39].

In a validation of the decisional procrastination scale, tension during decision making and discomfort about postponing tasks were independent of each other, suggesting that procrastination is not a unidimensional construct [40]. A study with university students found decisional procrastination was related to task failure, inciting anger, and rejection of high-spirited others [41]. Interpersonal dependency and self-defeating tendencies predicted decisional procrastination, suggesting indecision may become dispositional for individuals prone to codependency [41]. Ferrari (1993) found there is ecological validity to both trait and situational explanations of procrastination [42]. Some participants explained their procrastination as resulting from personal attributes, such as lack of energy and task aversion, while others gave situational explanations, such as having work or social commitments [42]. The literature suggests indecision manifests as both a trait and situational construct, and decisional procrastination has negative impacts on both performance and personality [43]. In clutter research, decisional procrastination is considered as a possible barrier to engaging in regular decluttering [44].

In a study considering characterological and contextual variables around indecision, decisional procrastinators reported high amounts of clutter, which affected quality of life [43]. Another study of U.S. workers found that indecision was related to higher amounts of office clutter, which was related to physical stress [45]. Evidently, indecision contributes to clutter-related outcomes, often predicting negative impacts of clutter on well-being.

Action–state orientation theory considers individual differences in approaching a task [46]. The action control scale measures goal-striving behavior across several subscales, one being hesitation to initiate difficult tasks vs decision-related action [47]. The construct measures cognition and behavior in task completion, considering the ability to make decisions, commit to a course of action, initiate action, avoid procrastination, manage competing demands, work towards challenging goals, and persist despite failure or setbacks [47]. Theoretically, action-oriented individuals initiate difficult tasks readily, and state-oriented individuals are more hesitant to engage in high-demand cognitive tasks [48]. Although many researchers described goal orientation as a disposition or facet of personality, other researchers argued a trait perspective fails to parsimoniously explain the process-oriented nature of goal setting [49,50].

Applied to personal projects, specifically decluttering projects, it may be beneficial to view action–state orientation as a situational phenomenon as opposed to a fixed personality trait. Persons struggling to manage their clutter have difficulty executing their personal projects, but that does not imply they struggle to set and achieve goals across all areas of their lives. Altering one’s behavior, resisting temptations, and changing how one feels requires self-regulation [51]. The set of neurocognitive skills required to solve a problem through self-regulation is referred to as executive function [52]. In relation to the self, executive function is ultimately responsible for the deliberate planning and intentional execution of individual behaviors, ranging from personal hygiene to personal projects [51,53]. Factors such as indecision and hesitation to initiate challenging tasks may impede one’s ability to set and execute more demanding tasks, such as decluttering an entire room.

“Executive function disorder” and other deviations from “normal” executive function are studied mostly in children with learning or developmental deficits and in older adults who may be losing the ability to care for themselves [54,55,56]. As a result, very few studies have assessed how executive dysfunction may manifest outside of these groups. Going beyond the need for executive function in goal-setting, Deci and Ryan’s (2000) self-determination theory posits three psychological needs that drive the pursuit of goals: competence, relatedness, and autonomy. Satisfying these needs is associated with well-being and intrinsic motivation [57]. When expectations for behavior are not internalized and transformed into intrinsic motivation, they are dependent on external contingencies (i.e., reactions from family members) [58]. Clutter experts suggest that when motives for behavior are not self-determined, decluttering tasks are more difficult to accomplish [2]. Thus, it is hypothesized that differences in reactance, indecision, and hesitation will partially explain decluttering outcomes.

The present study examined the role of psychological reactance, indecision (decisional procrastination), and hesitation to initiate goal-directed tasks as they relate to quality of life. It is hypothesized that a three-class model will be supported by a latent-class analysis. One decluttering type will emerge as low in indecisiveness, hesitation, and reactance, which will be related to more positive decluttering outcomes. A second decluttering type will consist of indecisive, hesitant, and possibly reactant individuals who struggle to manage their possessions at times, but do not have concerning issues related to clutter. Finally, a third group will emerge that is likely to experience indecision, hesitation, and reactance, which will be related to worse decluttering outcomes.

## 2. Materials and Method

Participants were recruited with the assistance of the Institute for Challenging Disorganization, an association of organizational coaches and decluttering professionals. The organization offers subscriptions to webinars, courses, resources, and a global network of professionals for members. Members encourage their clients, who are people who struggle with managing clutter, to participate in research conducted by ICD. The survey was created on Qualtrics and was disseminated to subscribers of ICD who forwarded the survey to their clients who indicated they are considering a decluttering project at the start of 2020. Thus, most participants were clients of organizing professionals. The survey took, on average, 20 min to complete.

### 2.1. Hypotheses

It is hypothesized that three classes of decluttering types may emerge. Action-oriented, non-reactant, decisive individuals with a lower impact of clutter on their lives will emerge as one group. This “declutterers” group may execute decluttering tasks regularly. Inversely, highly hesitant (state-oriented), reactant, and indecisive persons with high impacts of clutter on their lives will emerge as “non-declutterers”. Finally, a third group may emerge with individuals who are just as likely to be reactant, indecisive, and hesitant as they are unlikely. This group manages their clutter for the most part but may experience trouble decluttering at times.

### 2.2. Participants

A total of 227 participants completed the study. The self-reported genders of the participants were female (92.5%, *n* = 210), male (*n* = 13), and non-binary (*n* = 4). After excluding 2 participants who did not self-identify their gender, the final sample was 225 participants. The median age of respondents was 49.9 years old (*SD* = 12.4). Most participants self-identified as White (*n* = 198, 86%), Black or African American (*n* = 5, 2.2%), Asian or Pacific Islander (*n* = 3, 1.3%), Hispanic or Latinx (*n* = 18, 7.5%), American Indian or Alaskan Native (*n* = 2, 0.9%), Middle Eastern or North African (*n* = 0, 0%), and other (*n* = 6, 2.6%). The total percentage of race and ethnicity is greater than 100 because participants selected all options that applied. Most participants earned at least a high school diploma (*n* = 222, 99.5%), and well over half of participants earned at least a bachelor’s degree (*n* = 157, 68.4%) and had a mean personal income of $147,786.

### 2.3. Psychometric Scales

Clutter quality of life. All participants completed the 18-item Clutter Quality of Life Scale (CQLS; Roster et al. 2016) assessing the negative impact of clutter on one’s life [5]. Participants reported the extent to which they agree with each statement on a 7-point scale (1 = Strongly disagree; 7 = Strongly agree). Sample items include the following: I feel guilty when I think about the clutter in my home, my family has suffered as a result of the clutter in my home, and I can’t find things when I need them because of the clutter.

The index has four dimensions measuring the livability of one’s home and emotional, social, and financial implications of clutter. The livability of one’s home dimension contains five items and included items such as I have to move things in order to accomplish takes in my home and I have to be careful when walking through my home in order to avoid tripping over objects. The emotional dimension subscale included six items, such as I feel depressed by the clutter in my home and I’m worried about the amount of clutter in my home. The social dimension of CQLS includes four items, including I avoid having people come to my home because of clutter and I don’t have family members over as much as I would like because of the clutter in my home. The 3-item financial dimension of CQLS includes items such as I often buy things I already have because I don’t know where things are in my home and I have incurred debt I can’t really afford as a result of having too many possessions. The four dimensions discussed above may capture the broad constructs measured, but they have not been validated as subscales and, therefore, cannot be used for analytic purposes. Instead, a total impact score of clutter on quality of life equal to the mean of the 18 items is used. Roster and colleagues (2016) reported a coefficient alpha of 0.96 for the entire scale [5]. Swanson and Ferrari (2022) reported a coefficient alpha of 0.97 and a mean score of 4.26 [11]. As coefficient alpha approaches 1, the internal reliability of a scale increases. Because coefficient alpha is easy to interpret, it is widely used as a confirmation of scale reliability, although other practices, such as reporting structural equation modeling (SEM) estimates of reliability, are recommended [59,60]. For the purposes of exploratory analyses, SEM estimates were not calculated for each construct, which may limit the interpretability of the results for the present study.

The Clutter Quality of Life scale is used as a subjective measure of clutter and is distinct from clinical measures of hoarding, which use more objective assessments of accumulation and failure to dispose [2]. Although subjective perceptions of clutter may vary from objective measurements, subjective clutter scores were strongly correlated with subjective clutter scores in a recent study, suggesting that most people rate the impact of their clutter congruently with objective ratings [6].

Indecision. All participants completed the 5-item, unidimensional Decisional Procrastination [37,61]. Participants indicated the extent to which they engaged in various forms of cognitive procrastination, called indecision [39]. Sample items include I delay making decisions until it’s too late, and I don’t make decisions unless I really have to, each rated along a 5-point scale (1 = not true of me; 5 = totally true of me). Across a number of studies with adult samples, DP scores maintained a good reliability (Cronbach’s *α* ≥ 0.70) for the total scale score [39,62].

The Decisional Procrastination scale was validated in a study of university students and with older adults, demonstrating acceptable reliability (Cronbach’s *α* ≥ 0.70) [63]. Ferrari and colleagues reported Cronbach’s *α* = 0.92 [9].

Psychological reactance. The Hong Psychological Reactance Scale (HPRS; Hong & Faedda, 1996) is a 14-item scale measuring trait propensity to experience psychological reactance [64]. Sample items include I find contradicting others stimulating and Advice and recommendations induce me to do just the opposite. Participants reported the degree to which they agree with each item on a 5-point scale (1 = strongly disagree; 5 = strongly agree). In a study with an American sample (Shen & Dillard, 2005), the 14-item version of the scale maintained acceptable reliability (Cronbach’s *α* = 0.77) [65]. Psychological reactance was strongly correlated to several converging constructs, such as trait-anger (0.38) and depression (0.15). The scale maintained strong test–retest reliability in a 2-week (0.89) and 6-week (0.73) retest in an Australian sample. 

The scale has four subscales, which were confirmed by two principal components analyses: emotional response toward restricted choice, reactance to compliance, resisting influence from others, and reactance toward advice and recommendations [64,66]. In multivariate analyses of Hong’s reactance scale using nationally representative samples of Australian participants, four-factor structures emerged in each analysis [64,66]. The reliability coefficients of the total scale were 0.81 and 0.80 in the two studies, respectively. In a more recent confirmatory factor analysis [67], using an American sample, the subscale mean scores, standard deviations, and Cronbach’s alpha values were reported to be the following: emotional response toward restricted choice (*M* = 11.4, *SD* = 2.5, α = 0.63), reactance to compliance (*M* = 8.0, *SD* = 2.4, α = 0.57), resisting influence from others (*M* = 9.4, *SD* = 2.3, α = 0.53), and reactance toward advice and recommendations (*M* = 4.3, *SD* = 1.4, α = 0.48) [68]. The total reliability of the scale was not reported. Notably, the coefficient alphas for the four subscales of Hong’s psychological reactance scale fail to meet the conventional 0.70 cutoff for acceptable reliability. The results from the confirmatory factor analysis suggested that the four factors are correlated, demonstrating inter-item reliability. The authors recommend computing and interpreting scores on the basis of each factor of reactance, but previous studies are not consistent with these results. 

The present sample report the following mean scores, standard deviations, and Cronbach’s alpha values: emotional response toward restricted choice (*M* = 15, SD = 3.2, α = 0.69), reactance to compliance (*M* = 10.1, *SD* = 3.4, α = 0.71), resisting influence from others (*M* = 12.1, *SD* = 3.4, α = 0.70), and reactance toward advice and recommendations (*M* = 4.7, *SD* = 1.9, α = 0.68). The means, standard deviations, and Cronbach’s alpha values of the dimensions are included to better understand the experience of reactance in the samples in which it is studied. For the purposes of conducting a latent class analysis, the total for the entire scale is used. The four dimensions are outlined below but are not considered in the present study. 

The emotional response toward restricted choice subscale includes items such as The thought of being dependent on others aggravates me and I become frustrated when I am unable to make free and independent decisions. The reactance to compliance subscale includes items such as Regulations trigger a sense of resistance in me and I find contradicting others stimulating. The resisting influence from others scale includes items such as I am content only when I am acting of my own free will and When someone forces me to do something, I feel like doing the opposite. The reactance to advice and recommendations subscale includes the items I consider advice from others to be an intrusion and Advice and recommendations induce me to do just the opposite.

Hesitation. Action–state orientation theory measures individual differences in goal-striving behavior across several subscales, one being hesitation to initiate difficult tasks [47]. Action-oriented individuals initiate difficult tasks readily and state-oriented individuals are more hesitant to engage in goal-directed behavior. Sample items include When I know I must finish something soon: (1) I have to push myself to get started or (2) I find it easy to get it done and over with and When I have work to do at home: (1) It is often hard for me to get the work done or (2) I usually get it done right away. 

The mean score for each target scale was calculated using SPSS. The mean scores, standard deviation, correlation coefficients between all psychometric variables, and the scales’ Cronbach’s alpha are shown in Table 1. Using the median score for each target scale, participants were categorized into one of two groups across all variables. Participants were aggregated into groups for the purpose of conducting a latent class analysis. 

## 3. Results

**Figure 1:**
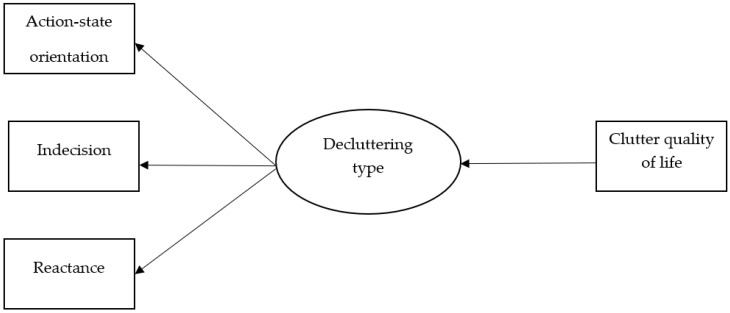
Latent Class Model.

A latent class analysis (LCA) examined indecision, reactance, and hesitation to assess whether subgroups of decluttering types emerged. Clutter quality of life was considered as a predictor of latent class membership. Figure 1 shows the latent model proposed in the present study. LCA aims to identify classification of individuals that are related to manifest indicators in probabilistic terms [68]. LCA is particularly useful when studying heterogenous populations consisting of unidentified groups that behave differently regarding the problem at hand [69]. The only assumption of LCA is that data are categorical. Participants were categorized into mutually exclusive groups across the three variables, described as either being ‘low’ or ‘high’ in each trait. No assumptions are made about linearity, normality of the distributions, or homogeneity of variance [69]. A two-class model and three-class model were compared across statistical and substantive criteria to determine which model had the best fit.

Class enumeration, or the determination of the appropriate number of latent classes for a given population, involves consideration of multiple statical criteria [70]. Experts recommend using the classification table based on class probabilities, the Akaike Information Criterion (AIC), the Bayesian Information Criterion (BIC), and other statistical criteria as necessary to ascertain the best model fit [70]. Lower BICs and AICs indicate better fit. For the present study, the two-class model yielded lower BIC and AIC values (AIC = 752.12, BIC = 801.45) than the three-class model (AIC = 782.32, BIC = 826.73). Goodness of fit measurements for both models are shown in Table 2.

Next, the average latent class posterior possibility, the average probability of the class model accurately predicting class membership, was reviewed [71]. The closer the diagonal values are to 1.0, the higher the probability of being assigned to a class given the case’s score on indicator variables. Using a cutoff of 0.80 to 0.90 for acceptable diagonal probabilities is recommended, although this requirement is less important when other criteria are met. The computed values for probabilities for class membership in the three-class model were closer to 1.0 across the three variables, while the two-class model failed to meet the 0.80 cutoff value for the reactance variable by a larger margin than the three-class model. Conditional probabilities and proportion estimates are shown in Table 3.

From a substantive perspective, a three-class model seems more plausible than a two-class model. A two-class model suggests that cognitive processes occur as binary and that participants either struggle to make decisions around clutter or they do not. Although the two-class model returned lower AIC and BIC values, it imposes a limitation on interpretability such that participants are described as being highly indecisive, reactant, and hesitant or none of those. Alternatively, the three-class model offers a more nuanced explanation of the target variables’ relationship to clutter quality of life. Examining the latent class posterior probabilities and considering the interpretation of a two-class model, a three-class model appeared more appropriate.

Results from the LCA supported the hypothesis that suggested that classes of decluttering-types existed amongst a population of people who are struggling to manage their clutter. Individual-level factors, such as tendency to procrastinate decisions, hesitation to initiate tasks, and reactance to advice, may inhibit decluttering despite existing motivation to do so. Class 1 consisted of participants who scored low on indecision and hesitation and were equally likely to be reactant or not. Class 1 represented 28% of the overall sample and will be referred to as the Efficient decluttering group. This decluttering type may experience some cognitive barriers to decluttering but is able to execute goal-oriented tasks with little or no obstacles. Class 2 appeared to consist of participants who were less likely to be procrastinators, and equally likely to be reactant and hesitant. This class of participants represented 38% of the sample and will be referred to as the Hesitant declutterers. Hesitant declutterers may face cognitive challenges when approaching goal-orientation tasks, but they are not indecisive and, thus, execute most decluttering tasks, minimizing the impact of clutter on quality of life. Finally, a third class of participants represented 33% of the overall sample. Participants in Class 3 are likely to be highly indecisive and hesitant and more likely to be reactant than the Resistant declutterers. Class 3 will be referred to as the Deterred declutterers; their experience of cognitive barriers may create persistent and strong negative impacts of clutter on quality of life. 

According to the regression model for a three-class model, as the impact of clutter became higher, participants were more likely to belong to a higher class or a class that experiences more indecision, reactance, and hesitation. Coefficients confirmed that moving from the Efficient group to the Hesitant group, clutter quality of life is expected to increase. Similarly, clutter quality of life is expected to increase from the Hesitant group to the Deterred group. Higher scores on clutter quality of life indicate a higher impact of clutter on quality of life, suggesting a negative effect of belonging to the Hesitant or Deterred groups.

The aim of this study was to examine latent, cognitive constructs in a community sample of participants considering a decluttering project. A latent class analysis examined three variables—indecision or decisional procrastination, hesitation to initiate challenging tasks, and psychological reactance—and their relationship to quality of life because of clutter. The results yielded a three-class model in which distinct decluttering types emerged as a function of the cognitive variables of interest.

Overall, the model classified participants into classes almost evenly, although the largest proportion of participants (39%) was classified into the group that had the highest (most negative) impact of clutter on quality of life. As clutter quality of life increased, participants were more likely to belong to Class 3, which was described by high indecision and high hesitance, followed by Class 2, which was described by moderate to high indecision, hesitance, and reactance. Finally, Class 1 was described as scoring low on all constructs, suggesting little or no interference with executing goal-oriented behavior such as decluttering.

## 4. Discussion

In this exploratory study, three cognitive variables were examined as they related to clutter related outcomes. Results provide substantial evidence for a three-class latent class model, suggesting barriers to decluttering outside of indecision. Decluttering involves clearing out messes, reducing disorder, and organizing [72]. Planning and executing decluttering projects require sequential decision-making, a skill which can be impaired, influencing acquisition, discarding, and maintenance of clutter [73]. On one end, people consistently struggle to manage their personal possessions and develop issues related to overaccumulation, eventually meeting criteria for hoarding disorder [16]. Many people, however, approach decluttering tasks regularly and manage personal possessions through self-regulatory processes.

While high reactance did not emerge in any of the decluttering groups, future studies should continue to examine the role of motivation and threats to the self as they relate to clutter. Both Hesitant and Deterred decluttering groups were prone to experiencing reactance. A situation in which hesitation and indecision exacerbate the perception of a threat is plausible, implying differences in individual propensity to reactance. For example, a hesitant and indecisive person may avoid a major decluttering task for so long that another household member undertakes part of the task. Under these conditions, the individual’s proneness to experiencing threat and consequent reactance may moderate how the external pressure to start the task is perceived. If the person is open to receiving help and does not feel threatened, reactance may not affect decluttering and the external pressure may lead to a positive decluttering outcome. If the person feels a threat to the self from the external pressures, they may work to restore the behavioral freedom by stopping or reversing decluttering efforts. This is where cultural context might play a role: For people with more interdependent self-construal in a more collectivistic cultural setting, decluttering advice from a close other will likely cause less reactance than for someone with a more individualistic sense of self, who might be more prone to perceiving such advice as an external pressure [22,23].

Hesitation to initiate challenging tasks, or action–state orientation, appears to play a crucial role in decluttering projects, with both the Hesitant and Deterred groups experiencing negative consequences of their cognitions on clutter outcomes. Interventionists may infer that those who struggle with clutter are not simply indecisive and that indecision may cause spillover effects such as hesitation. The opposite is also possible from a trait perspective, with state-orientation making individuals prone to indecision. Considering a bidirectional relationship between these variables may strengthen interventive and preventative solutions to managing clutter. Mitigating maladaptive decision-making strategies, such as procrastination, improving self-regulation processes to reduce overaccumulation, and teaching goal setting and goal executing skills, offers a comprehensive solution to helping those struggling with clutter. In other words, organizing professionals should consider how they can not only help their clients manage specific clutter-related problems but prevent clutter from becoming a persistent problem through better understanding of the individual’s consumption behavior.

Purchasing items usually occurs to meet utilitarian needs; humans need to consume some amount of material goods for survival [74]. More occasionally, shopping is a pastime and may serve a means of managing emotions or to express and establish an identity [75,76,77]. When a possession is no longer useful, consumers must decide to either manage the item or dispose of it. At the end of a product’s life cycle, they make attributions about the disposability of the item or the ways in which it can be disposed of, its longevity, their experience with using it, their attachment to it, and more [78]. These reflections at the decluttering stage may inform future consumption behavior and should be explored by consumer psychologists and organization professionals working with people experiencing clutter-related problems, especially in indecisive populations.

In 2009, 6% of the U.S. population was afflicted by compulsive buying, which is more than double the percentage of Americans who experience symptoms of hoarding [74]. The discrepancy suggests that while Americans may be consuming to the extent of experiencing clutter-related problems, they may not perceive their behavior as overconsumption. Furthermore, because researching overconsumption may come at a cost to the global economy, it is overpowered by investment in market research and advertising. In other words, the rate of innovation and the application of psychology to sell products may overshadow efforts to criticize our consumption patterns. The present study provides a first glance at how a climate of overconsumption and our orientation towards complex tasks, such as decluttering, may interact.

Contextually, several factors may provide a partial explanation for the results obtained. For many, the early weeks of the pandemic consisted of a sedentary lifestyle. Venues for socializing, such as restaurants, sporting arenas, and parks, remained closed, confining Americans to their homes. A meta-analysis including 23 studies of dietary behaviors and weight loss/gain showed an increase in the number of snacks consumed in ten studies, with another eleven studies reporting desired changes in eating habits with the increased ability to cook at home [79]. In contrast, nine studies found a reduction in fresh produce, suggesting that access to food and food choices may vary depending on the sample observed. The initial weeks of the pandemic were also marked by panic buying, a pattern of overbuying intended to mitigate the possible scarcity of products [80]. From this handful of consumer studies, a shift in decision-making is observed on a larger scale. Known associations among clutter-related problems, binge-eating, and other types of overconsumption prompt inquiry regarding how environmental disasters affect decision-making. 

There are some limitations to this study. The participants included may be considered a convenience sample. Because participants subscribe to an organization that provides decluttering solutions, it is possible that this sample may not represent the national population who do not seek help with organizing. Aggregating participants into groups on the basis of median cutoffs may have controlled for this limitation in part, but it is nonetheless important to consider a broader sample in future research. Another limitation is the demographic composition of participants; most were older White women, which is unrepresentative of the national population. Clutter researchers and organizing professionals alike suspect that gender role endorsement affects the responsibilities people assume in home settings, one of which is maintaining spaces through organization. Future studies should aim to recruit a sample that represents a broader spectrum of gender and includes participants from different ethnicities and races.

Exploratory research such as the present study generates partial explanations for behavior that should be explored further in experimental research. From these results, we observed differences in cognitive processes in a large, heterogenous sample of participants who at some point struggled with managing clutter. For psychologists and organizational professionals, the study contributes to a greater understanding of the phenomenon that hinder or prevent their clients from engaging in regular decluttering tasks. Clutter research should continue to expand its considerations for cognitive processes related to goal-setting.

## 5. Conclusions

Clutter management relies on cognitive processes such as decision-making and action-orientation toward decluttering tasks. In some persons, these capacities may be reduced and consequentially affect quality of life because of clutter. Indecision is a well-researched concept as it relates to decluttering, yet other psychological constructs affecting decluttering behavior remain largely unexplored. Those struggling to manage their personal possessions and the practitioners who provide supportive services might consider the role of indecision, reactance, and their overall disposition towards difficult tasks to better understand decluttering barriers and inform strategies for management.

## Figures and Tables

**Table 1:** Mean, Standard Deviation, Cronbach’s alpha, and Correlations between Psychometric Variables.

	*M*	*SD*	1	2	3	4
1. Clutter Quality of Life	3.73	1.77	0.97			
2. Indecision	1.9	1.06	0.62 **	0.91		
3. Reactance	3.00	0.70	0.28 **	0.29 **	0.87	
4. Hesitation	1.34	0.29	0.68 **	0.69 **	0.33 **	0.86

Note: ** denotes significance at *p* < 0.01. Cronbach’s alpha is presented on the diagonal.

**Table 2:** Goodness of fit measurements for 2-class and 3-class models.

	Number of Classes	Log- Likelihood	AIC	BIC
Latent variable	2	−368.58	752.12	801.45
	3	−368.58	782.32	826.73

**Table 3:** Conditional probabilities and proportion estimates.

	Class 1 (53.6%)	Class 2 (46.4%)	
Procrastination	0.18	0.91	
Reactance	0.43	0.57	
Hesitation	0.18	1	
	Class 1 (28.3%)	Class 2 (38.4%)	Class 3 (33.3%)
Procrastination	0.1	0.35	1
Reactance	0.29	0.51	0.59
Hesitation	0	0.53	1

## Data Availability

Restrictions apply to the availability of these data. Data were obtained from Catherine Roster in collaboration with the Institute of Challenging Disorganization and are available from the author of this article with the permission of both parties.
